# LncRNA NEAT1 promotes gastric cancer progression via miR-1294/AKT1 axis

**DOI:** 10.1515/med-2020-0218

**Published:** 2020-10-09

**Authors:** Dianchao Wu, Hui Li, Junfeng Wang, Hua Li, Qihai Xiao, Xiaofeng Zhao, Zhibin Huo

**Affiliations:** Department of Surgical Oncology, Xingtai People’s Hospital, No.16, Hongxing Street, Xingtai, 054031, Hebei, China; Department of Colorectal Surgery, Tianjin Medical University Cancer Institute and Hospital, Tianjin, China

**Keywords:** NEAT1, gastric cancer, miR-1294, AKT1, PI3K/AKT/mTOR pathway

## Abstract

Long non-coding RNAs (lncRNAs) were reported to promote the development of gastric cancer (GC). Nuclear-enriched abundant transcript 1 (NEAT1) played a great role in diverse cancers, but the mechanism of NEAT1 in GC remains indistinct. NEAT1 and AKT1 were distinctly up-regulated and miR-1294 was down-regulated in GC tissues and cells. Cell proliferation and metastasis were refrained but apoptosis was promoted in GC cells after knockdown of NEAT1. NEAT1 negatively regulated miR-1294 expression, and the miR-1294 inhibitor reverted the si-NEAT1-induced effect on GC cells. NEAT1 modulated AKT1 expression through miR-1294, and the si-NEAT1-induced effect was relieved by AKT1. NEAT1 affected phosphatidylinositol 3-kinase (PI3K)/AKT/mammalian target of rapamycin (mTOR) signaling pathway via regulating miR-1294 and AKT1. NEAT1 could modulate cell proliferation, apoptosis, and metastasis in GC cells by regulating the PI3K/AKT/mTOR signaling pathway via the miR-1294/AKT1 axis, showing the great potential for NEAT1 as a valid biomarker in the progression and treatment of GC.

## 1 Introduction

Gastric cancer (GC) is regarded as the third reason for death caused by tumors [25]. As a heterogeneous disease, the risk factors of GC cover different aspects, including age, *Helicobacter pylori*, familial syndromes, tobacco, alcohol, and diet [15]. Although some precancerous lesions can be screened by radiography, endoscopy, and serology [15], most patients with GC have been diagnosed at an advanced stage because of the unobservable symptoms. The 5-year survival of patients with GC is averagely in 25–30% [2]. Thus, it is imminently required to explore the function mechanism underlying GC and to find more available diagnostic or therapeutic targets.

Long non-coding RNAs (lncRNAs) are longer than 200 nucleotides and lose the protein-coding ability due to the incomplete open reading frames [16]. In recent years, numerous reports have declared that lncRNAs participated in regulating the biological processes of GC as crucial regulators [7,23]. For example, GMAN was up-regulated in GC tissues and heightened cell metastatic ability [38] and TUG1 enhanced cell proliferation and invasion in GC [19]. Li et al. found that lncRNA M26317 was associated with GC development and prognosis [14]. Nuclear-enriched abundant transcript 1 (NEAT1) was reported to exert great function in different cancers [5], including as an oncogene in GC [3]. Here, we aimed at discovering a specific functional mechanism of NEAT1 in GC.

LncRNAs have a “sponge-like” effect on specific microRNAs (miRNAs), which then combined with 3′-untranslated regions (3′-UTRs) of the target gene to further regulate the evolution of diverse human cancers [26]. For instance, AWPPH played an accelerative role in the progression of non-small cell lung cancer by the up-regulation of CDK6 via sponging miR-204 [31] while PVT1 facilitated colorectal cancer cell proliferation and invasion by sequestering miR-214-3p to affect the IRS1 level [21]. Previous studies have suggested that microRNA-1294 (miR-1294) was down-regulated in GC and inhibited the epithelial-mesenchymal transition process via targeting FOXK1 [22,30]. Serine/threonine kinase 1 (AKT1) belongs to the AKT family and has crucial modulation in multiple cancers [6,11]. Han et al. announced that AKT1 enhanced cell growth and cell cycle progression of GC [9]. It remains unclear whether NEAT1 had a sponge effect on miR-1294 to regulate AKT1 in GC.

AKT1 can be suppressed by phosphatase and tensin homolog deleted on chromosome 10 (PTEN) to result in the inhibition of phosphatidylinositol 3-kinase (PI3K)/AKT/mammalian target of rapamycin (mTOR) pathway in GC [20]. Ribosomal p70S6 kinase 1 (S6K1) is an mTOR complex 1-related protein, as an index of the activation of the mTOR pathway [1]. The relation of NEAT1 and miR-1294 with PI3K/AKT/mTOR pathway is unknown.

Thus, this study is devoted to affirming the target relation of NEAT1/miR-1294 and miR-1294/AKT1, then further researching the regulatory relation between NEAT1/miR-1294/AKT1 and PI3K/AKT/mTOR pathway in GC.

## 2 Materials and methods

### 2.1 Tissues collection and cell culture

Thirty pairs of GC tissues and adjoining normal tissues were acquired from patients going through gastrectomy in Xingtai People’s Hospital. They didn’t receive any treatment for GC before surgical excision. Acquired tissues were immediately frozen in a liquid nitrogen container. The written informed consent forms were signed by all patients. The study was approved by the Ethics Committee of Xingtai People’s Hospital.

Human GC cell lines (NCI-N87, HGC-27, MGC-803, and AGS) were bought from the Cell Resource Center of Shanghai Institute for Biological Sciences, Chinese Academy of Sciences (Shanghai, China), and normal gastric epithelial cell line GES-1 was purchased from COBIOER Biosciences Co., Ltd (Nanjing, China). AGS cells were cultivated in F12K medium and other cells were maintained in Roswell Park Memorial Institute-1640 (RPMI-1640; Invitrogen, Carlsbad, CA, USA) complemented with 10% fetal bovine serum (FBS; Invitrogen), 100 U/mL penicillin, and 100 µg/mL streptomycin (Invitrogen). All cells were grown in a 37°C humid incubator with 5% CO_2_.

### 2.2 Cell transfection

Small interfering RNA (siRNA) targeting NEAT1 (si-NEAT1), siRNA negative control (si-NC), miR-1294 inhibitor (anti-miR-1294), miR inhibitor control (anti-miR-NC), miR-1294 mimic (miR-1294), and miR mimic control (miR-NC) were synthesized from RIBOBIO (Guangzhou, China). NEAT1 overexpression plasmid (NEAT1) and AKT1 overexpression plasmid (AKT1) were constructed using pcDNA vectors (Invitrogen). Lipofectamine 2000 (Thermo Fisher Scientific, Waltham, MA, USA) was utilized to carry out cell transfection. First, cells were cultured to reach 70–90% monolayer confluence in a 24-well plate. Next, plasmid DNA–lipid complexes were prepared and incubated for 5 min at room temperature, then the complexes were added to cells. Finally, cells were replaced in the incubator to culture for the following experiments.

### 2.3 RNA extraction and quantitative real-time polymerase chain reaction (qRT-PCR)

Trizol reagent (Invitrogen) was used for the extraction of total RNA from GC tissues and cells. Complementary DNA (cDNA) was synthesized by Prime Script TM RT reagent kit (Takara, Shiga, Japan), following the user’s guideline. The PCR reaction was performed by SYBR^®^ Premix Ex Taq™ Kit (Takara) on the StepOnePlus Real-time PCR System (Thermo Fisher Scientific). NEAT1 and AKT1 were normalized by glyceraldehyde-3-phosphate dehydrogenase (GAPDH), and small nuclear RNA U6 was utilized to standardize miR-1294. The sequences of primers were shown below: NEAT1: forward 5′-GUCUGUGUGGAAGGAGGAATT-3′ and reverse 5′-UUCCUCCUUCCACACAGACTT-3′; miR-1294: forward 5′-TATGATCTCACCGAGTCCT-3′ and reverse 5′-CACCTTCCTAATCCTCAGTT-3′; AKT1: forward 5′-GCTGGACGATAGCTTGGA-3′ and reverse 5′-GATGACAGATAGCTGGTG-3′; GAPDH: forward 5′-TGCACCACCAACTGCTTAGC-3′ and reverse 5′- GGCATGCACTGTGGTCATGAG-3′; and U6: forward 5′-CTCGCTTCGGCAGCACA-3′ and reverse: 5′-AACGCTTCACGAATTTGCGT-3′. Collected data were calculated through the 2^−∆∆Ct^ approach.

### 2.4 3-(4,5-Dimethylthiazole-2-y1)-2,5-diphenyl tetrazolium bromide (MTT) assay

NCI-N87 and AGS cells were cultured overnight in a 96-well plate and transfected with plasmids. We selected three-time points 24, 48, and 72 h for detection. Cells were incubated with 10 µL MTT (Thermo Fisher Scientific) for 4 h. After supernatants were discarded, cells were incubated with 100 µL dimethylsulfoxide (DMSO; Thermo Fisher Scientific) for approximately 10 min. Then, optical density (OD) value at 490 nm was determined by a microplate reader (Thermo Fisher Scientific). Data were recorded and analyzed.

### 2.5 Flow cytometry

Cells were first digested by 0.25% trypsin (Invitrogen) without Ethylene Diamine Tetraacetic Acid (EDTA) and centrifuged for collection. Phosphate buffered saline (PBS) was precooled at 4°C for washing cells. Washed cells were resuspended with 1× binding buffer. Annexin V-fluorescein isothiocyanate (Annexin V-FITC) and propidium iodide (PI) kit (Sigma-Aldrich, Saint Louis, MO, USA) was utilized to colorate. Apoptosis cells were first labeled by Annexin V-FITC and then stained by PI. In the end, double-dyed apoptotic cells were observed under the flow cytometer (Becton Dickinson; San Jose, CA, USA).

### 2.6 Transwell migration and invasion assays

Transwell 24-well chamber (Corning Life Sciences, Corning, NY, USA) was used to detect cell metastasis. In invasion assay, the lower surface of the upper chamber was first enveloped with matrigel (Corning Life Sciences), whereas migration assay was not required. After NCI-N87 and AGS cells were digested and washed, cells suspension liquid was prepared using a serum-free medium and seeded into the upper chamber, and the corresponding medium with 10% FBS was added to the lower chamber. After incubation for 24 h, cells were fixed by 4% paraformaldehyde and stained by crystal violet (Sangon Biotech, Shanghai, China). The chamber was washed by water and sealed with neutral gum. Migrated and invaded cells were counted through a microscope.

### 2.7 Dual-luciferase reporter assay

The wild-type NEAT1 and 3′-UTR of wild-type AKT1 were inserted into pGL3 vectors (Promega, Madison, WI, USA) and named as WT-NEAT1 and AKT1 3′UTR-WT. Their mutant-type controls were also constructed as MUT-NEAT1 and AKT1 3′UTR-MUT. They were co-transfected into NCI-N87 and AGS cells with miR-1294 or miR-NC. First, the Passive Lysis Buffer (PLB; Promega) was diluted into 1× PLB. Then, cells in a 24-well plate were rinsed with 1× PBS and lysed using 1× PLB. The luciferase activity of firefly and renilla was examined by a dual-luciferase reporter system (Promega), following the manufacturer’s specification.

### 2.8 Western blot assay

Radio-Immunoprecipitation Assay (RIPA) lysis buffer with proteinase inhibitor (Sigma-Aldrich) was used for the extraction of total proteins from GC tissues and cells. To begin with, proteins were segregated with sodium dodecyl sulfate–polyacrylamide gel electrophoresis (SDS–PAGE) and transferred onto polyvinylidene fluoride (PVDF) membranes (Sigma-Aldrich). Next, PVDF membranes were blocked using 5% skim milk for more than 3 h, then incubated with primary antibodies overnight at 4°C. The primary antibodies used were presented as follows anti-AKT1 (1:1,000; ab81283, Abcam, Cambridge, United Kingdom), anti-PTEN (1:1,000; ab32199, Abcam), anti-phospho-AKT (p-AKT, T308; 1:1,000, ab38449, Abcam), anti-AKT (1:1,000; ab8805, Abcam), anti-S6K1 (1:1,000; ab32529, Abcam), and anti-GAPDH (1:3,000; ab9485, Abcam). Then under the condition of room temperature, specific goat anti-rabbit secondary antibody (1:5,000; ab205718, Abcam) was used to incubate the membranes for 1 h. Finally, PVDF membranes were colored through the enhanced chemiluminescence (ECL) reagent (Sigma-Aldrich), and the results were observed under the Image J software (NIH, Bethesda, MD, USA).

### 2.9 Statistical analysis

All experiments were independently repeated three times, and the results were revealed as the mean ± standard deviation (SD). Data were analyzed by SPSS 19.0 and processed by GraphPad Prism 7 software. Difference analysis was evaluated through Student’s *t*-test or one-way ANOVA followed by Tukey’s test. Spearman’s correlation coefficient was used to analyze the linear relationship between the levels of NEAT, miR-1294, and AKT1 in GC tissues. *P* < 0.05 was considered as statistically significant difference.

## 3 Results

### 3.1 NEAT1 was up-regulated in GC tissues and cells

**Figure 1:**
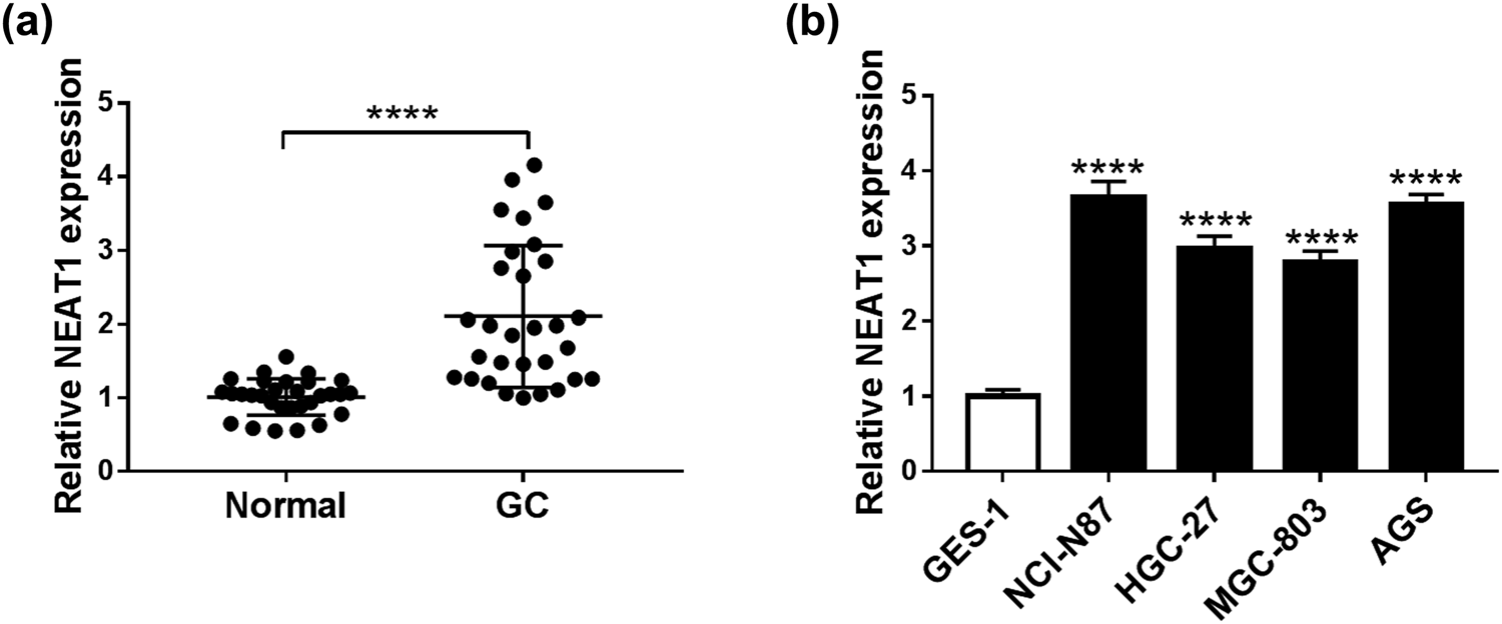
NEAT1 was up-regulated in GC tissues and cells. (a and b) QRT-PCR was utilized to examine the relative NEAT1 expression in GC tissues (a) and cells (b). ****P < 0.0001.

At first, qRT-PCR was used for the detection of NEAT1 in tissues and cells. The data indicated that NEAT1 expression was obviously higher in GC tissues than that in normal tissues (Figure 1a). And NEAT1 was signally up-regulated in GC cell lines (NCI-N87, HGC-27, MGC-803, and AGS), compared to the normal gastric epithelial cell line GES-1 (Figure 1b). The high expression of NEAT1 insinuated that NEAT1 might play a vital role in GC progression.

### 3.2 Knockdown of NEAT1 suppressed cell proliferation and metastasis, but motivated apoptosis in GC cells

**Figure 2:**
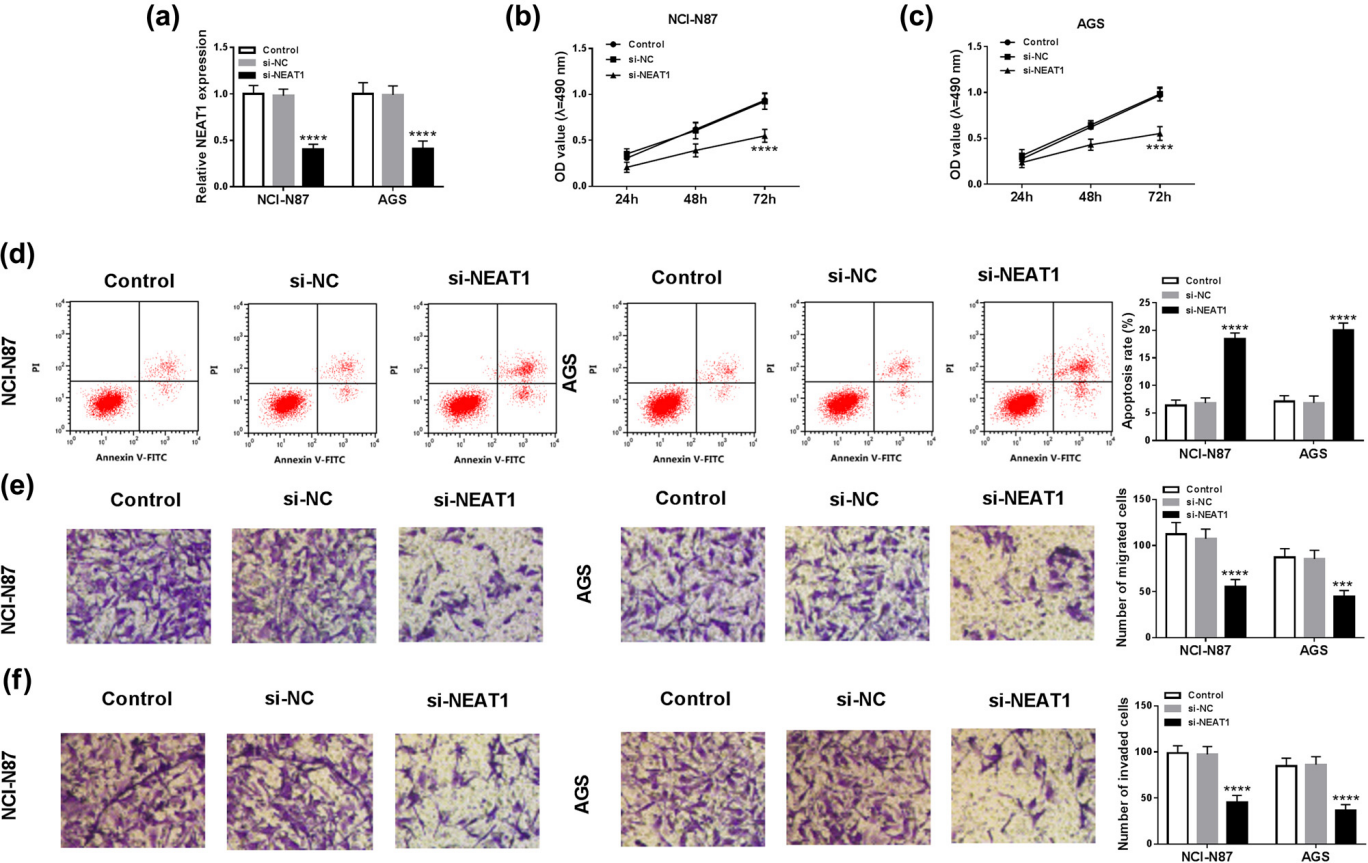
Knockdown of NEAT1 suppressed cell proliferation and metastasis, but motivated apoptosis in GC cells. (a) The knockdown effect of si-NEAT1 was assessed by qRT-PCR. (b and c) Cell proliferation was detected by MTT assay in NCI-N87 and AGS cells transfected with si-NEAT1. (d) Cell apoptosis was determined by flow cytometry. (e and f) Cell migration and invasion were examined by transwell assay. ***P < 0.001, ****P < 0.0001.

To research the function of NEAT1 in GC, NCI-N87 and AGS cells were selected for transfection with si-NEAT1. Compared with the blank control and si-NC groups, relative NEAT1 expression was overtly decreased in the si-NEAT1 group (Figure 2a), indicating the interfering effect was great. Then MTT assay presented that OD value was distinctly reduced in NCI-N87 and AGS cells transfected with si-NEAT1 (Figure 2b and c). Flow cytometry showed that the apoptosis rate was higher in the si-NEAT1 group than that in the control groups (Figure 2d). In addition, cell metastasis ability was assessed by transwell migration and invasion assays. As Figure 2e and f depicted, migrated and invaded cells were remarkably declined after transfection with si-NEAT1. These results demonstrated that cell proliferation and metastasis were inhibited but apoptosis was motivated after knockdown of NEAT1.

### 3.3 Down-regulation of miR-1294 relieved the si-NEAT1-induced effects on proliferation, apoptosis, and metastasis of GC cells

**Figure 3:**
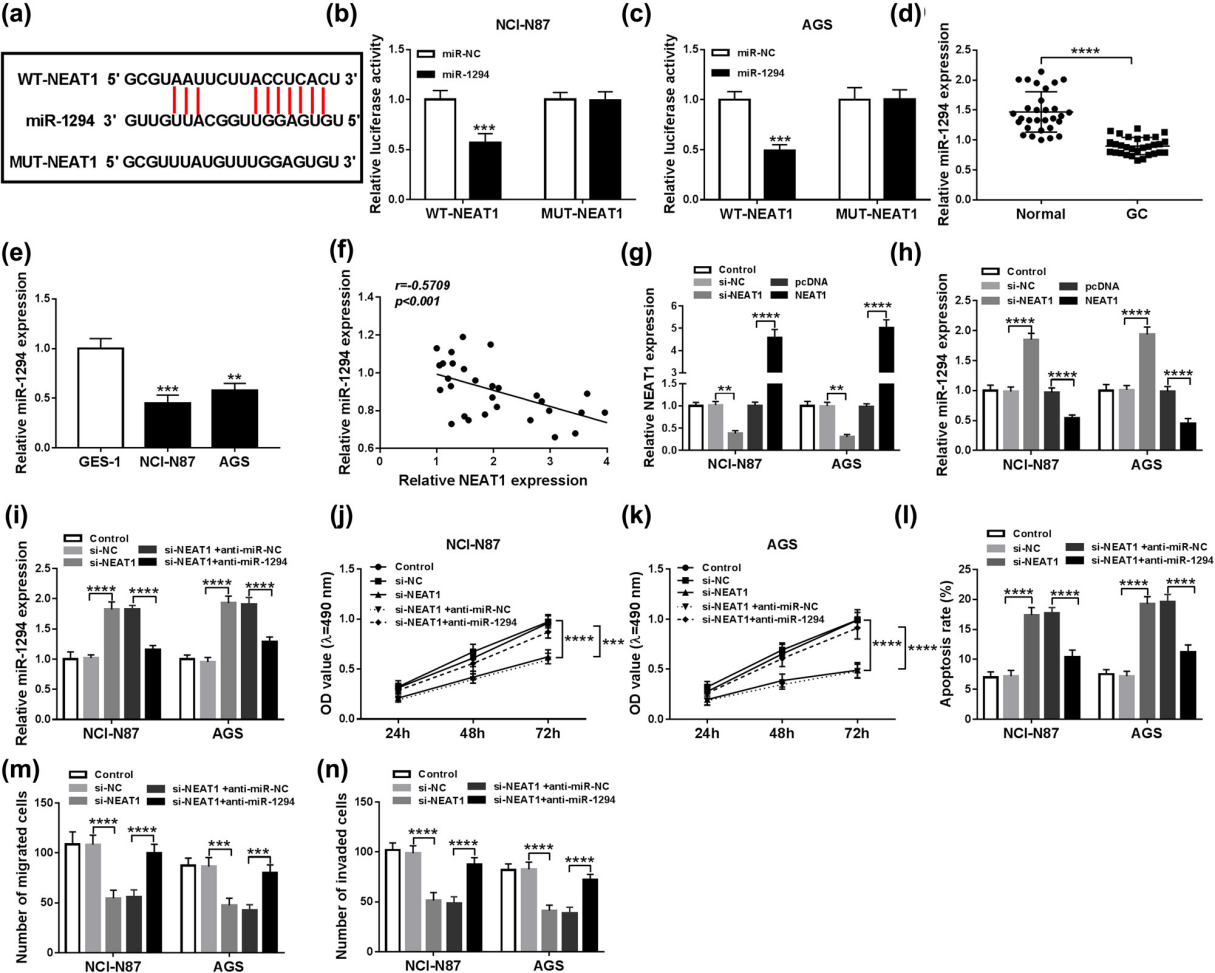
Down-regulation of miR-1294 relieved the si-NEAT1-induced effects on proliferation, apoptosis, and metastasis of GC cells. (a) The binding sites of NEAT1 and miR-1294 were predicted by Starbase v2.0. (b and c) Luciferase activity of NCI-N87 and AGS cells co-transfected with WT-NEAT1 or MUT-NEAT1 and miR-1294 or miR-NC was measured by dual-luciferase reporter assay. (d and e) MiR-1294 expression was examined by qRT-PCR in tissues and cells. (f) The linear relationship between NEAT1 and miR-1294 in GC tissues was analyzed by Spearman’s correlation coefficient. (g) QRT-PCR was used to detect the knockdown effect of si-NEAT1 and overexpressed effect of NEAT1. (h) The expression of miR-1294 was detected by qRT-PCR after transfection with si-NEAT1 or NEAT1. (i) QRT-PCR was used to determine the expression of miR-1294. (j and k) MTT was used to detect cell proliferation. (l) Flow cytometry was utilized to determine apoptotic cells. (m) Transwell assay was used to measure migrated and invaded cells. **P < 0.01, ***P < 0.001, ****P < 0.0001.

Starbase v2.0 was used for the prediction of targets. As shown in Figure 3a, WT-NEAT1 contained the binding sites of miR-1294. Dual-luciferase reporter assay indicated that luciferase activity of the WT-NEAT1 group was decreased by miR-1294, whereas there was no obvious change in the MUT-NEAT1 group in both NCI-N87 and AGS cells (Figure 3b and c), implying NEAT1 targeted miR-1294. Then qRT-PCR analysis showed that miR-1294 was evidently down-regulated in GC tissues (Figure 3d), and cell lines NCI-N87 and AGS (Figure 3e). Also, a significant negative correlation between the levels of NEAT1 and miR-1294 in tissues was observed (*r* = −0.5709, *P* < 0.001) (Figure 3f). QRT-PCR revealed that the knockdown effect of si-NEAT1 and overexpressed effect of NEAT1 were both marked (Figure 3g). In addition, knockdown of NEAT1 prominently increased the expression of miR-1294, while overexpression of NEAT1 reduced the expression (Figure 3h), indicating NEAT1 could modulate the level of miR-1294 in GC cells. And after transfection with anti-miR-1294, the si-NEAT1-induced increased expression of miR-1294 was strikingly descended (Figure 3i). Moreover, the si-NEAT1-induced inhibitory effect on the proliferation of NCI-N87 and AGS cells was alleviated by anti-miR-1294 (Figure 3j and k). The promoted effect on apoptosis induced by si-NEAT1 was also reverted after transfection with anti-miR-1294 (Figure 3l). Similarly, transwell assay revealed that anti-miR-1294 restored the si-NEAT1-induced effects on migration and invasion (Figure 3m). Taken together, miR-1294 was a target of NEAT1, and down-regulation of miR-1294 relieved the si-NEAT1-induced effect on GC cells.

### 3.4 NEAT1 elevated the expression of AKT1 by sponging miR-1294

**Figure 4:**
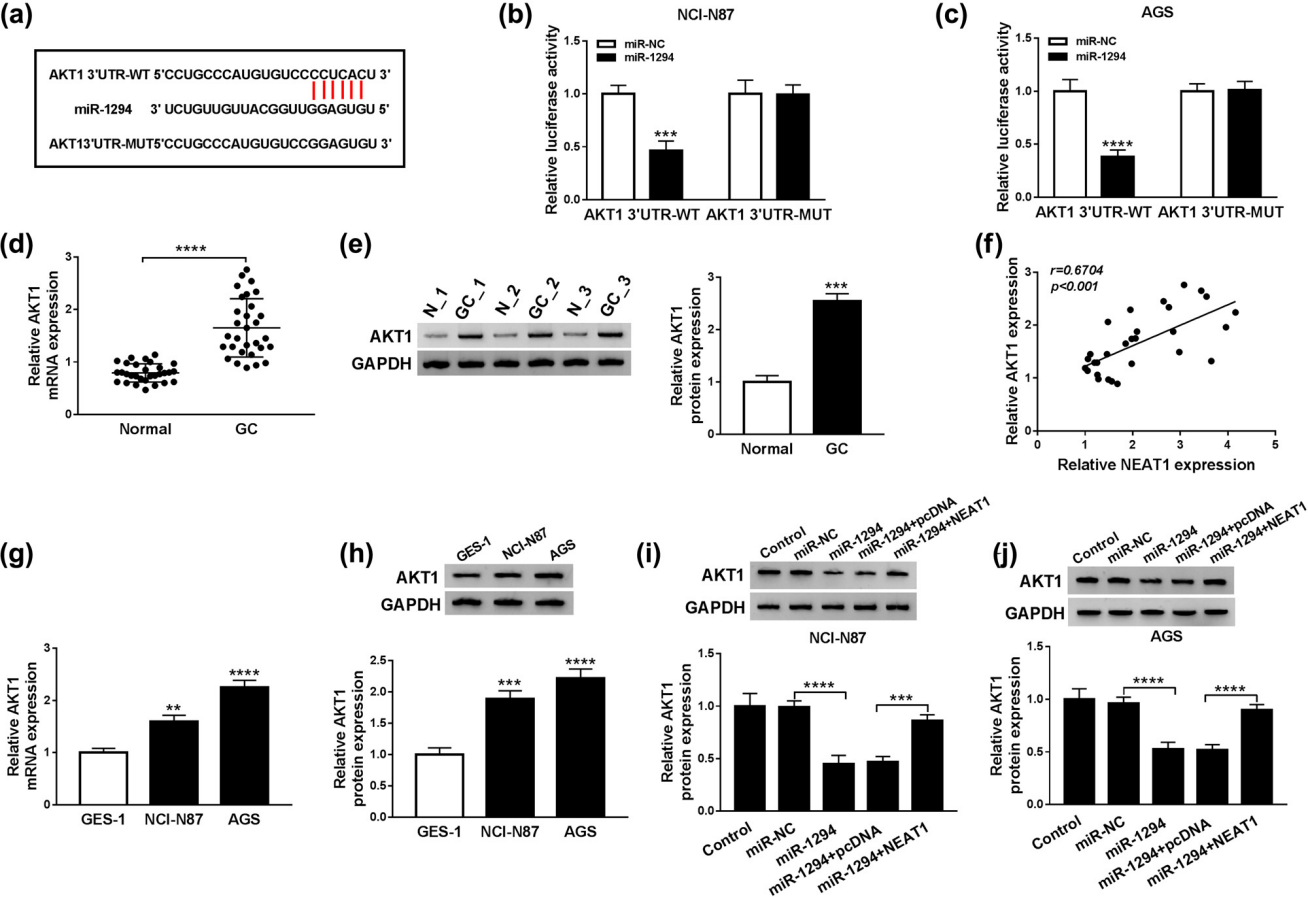
NEAT1 elevated the expression of AKT1 by sponging miR-1294. (a) Starbase v2.0 was used to predict the targets of miR-1294. (b and c) Dual-luciferase reporter assay was used to verify the relation of miR-1294 and AKT1. (d and e) The expression of AKT1 in GC tissues was detected by qRT-PCR and Western blot. (f) Spearman’s correlation coefficient was used to analyze the relation between miR-1294 and AKT1 in GC tissues. (g and h) AKT1 expression in GC cells was determined by qRT-PCR and Western blot. (i and j) Western blot was utilized to measure AKT1 protein expression in NCI-N87 and AGS cells transfected with miR-1294, miR-1294 + NEAT1, and relative controls. **P < 0.01, ***P < 0.001, ****P < 0.0001.

Based on Starbase v2.0, 3′-UTR of wide-type AKT1 with the binding sites of miR-1294 and its mutant-type without the binding sites were cloned into pGL3 vector (Figure 4a). Constructed AKT1 3′UTR-WT and AKT1 3′UTR-MUT were co-transfected into cells for dual-luciferase reporter assay. The results showed that luciferase activity of AKT1 3′UTR-WT group in NCI-N87 and AGS cells was markedly reduced by miR-1294, in comparison to the AKT1 3′UTR-MUT group (Figure 4b and c). Next, qRT-PCR and Western blot demonstrated that the levels of AKT1 mRNA and protein were prominently enhanced in GC tissues (Figure 4d and e). And the positive relation between the expression of NEAT1 and AKT1 in GC tissues was notable (*r* = 0.6704, *P* < 0.001) (Figure 4f). The levels of AKT1 mRNA and protein in GC cell lines NCI-N87 and AGS were also higher than these in normal GES-1 cells (Figure 4g and h). Moreover, Western blot indicated that miR-1294 inhibited the expression of AKT1 protein, while this inhibitory effect was abated after the overexpression of NEAT1 in NCI-N87 and AGS cells (Figure 4i and j). The above-mentioned results manifested that AKT1 was a target of miR-1294 and NEAT1 elevated AKT1 expression by sponging miR-1294 in GC cells.

### 3.5 Overexpression of AKT1 restored the si-NEAT1-induced effects on cell proliferation, apoptosis, and metastasis of GC cells

**Figure 5:**
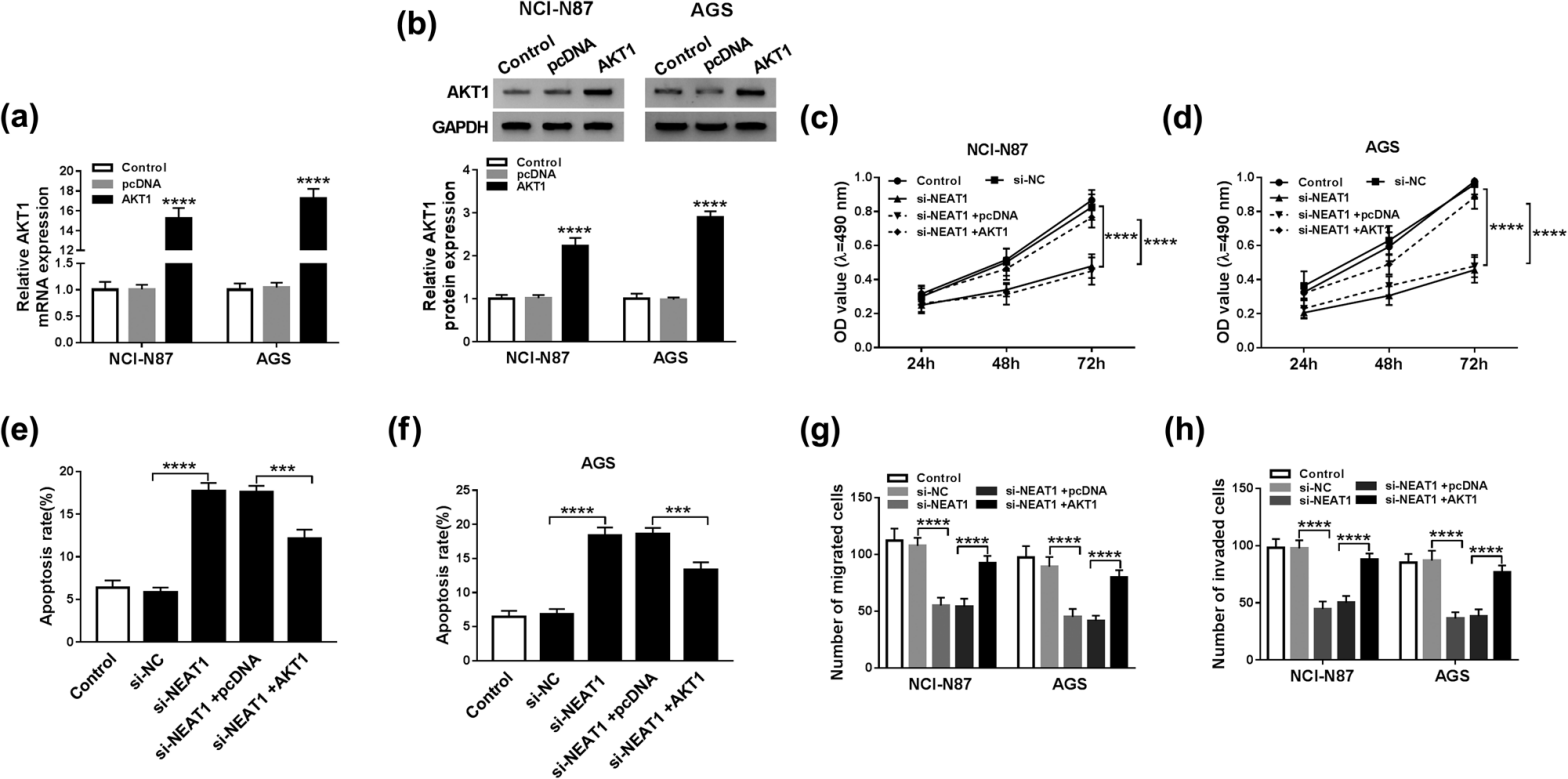
Overexpression of AKT1 restored the si-NEAT1-induced effects on cell proliferation, apoptosis, and metastasis of GC cells. (a and b) QRT-PCR and Western blot were used to measure the overexpressed effect of AKT1. (c and d) Cell proliferation in NCI-N87 and AGS cells transfected with si-NEAT1 and AKT1 was detected by MTT. (e and f) Cell apoptosis was determined by flow cytometry. (g and h) Migrated and invaded cells were examined by transwell assay. ***P < 0.001, ****P < 0.0001.

For further investigation of the regulatory mechanism between NEAT1 and AKT1, an AKT1 overexpression vector was constructed. QRT-PCR and Western blot analyzed that the levels of AKT1 mRNA and protein were dramatically elevated in NCI-N87 and AGS cells after transfection with AKT1 (Figure 5a and b). NCI-N87 and AGS cells were severally transfected with si-NEAT1, si-NEAT1 + AKT1, and relative controls. MTT reflected that the si-NEAT1-motivated effect on cell proliferation was mitigated by AKT1 overexpression (Figure 5c and d). Cell apoptosis was measured by flow cytometry. As presented in Figure 5e and f, AKT1 notably reduced the si-NEAT1-induced enhancive apoptosis rate. Besides, the reduction of migrated and invaded cells induced by si-NEAT1 was partly prevented by AKT1 overexpression. These results suggested that si-NEAT1-induced effects on cell proliferation, apoptosis, and metastasis were abated by AKT1 overexpression in GC cells.

### 3.6 Down-regulation of NEAT1 repressed PI3K/AKT/mTOR signaling pathway via elevating miR-1294 and decreasing AKT1

**Figure 6:**
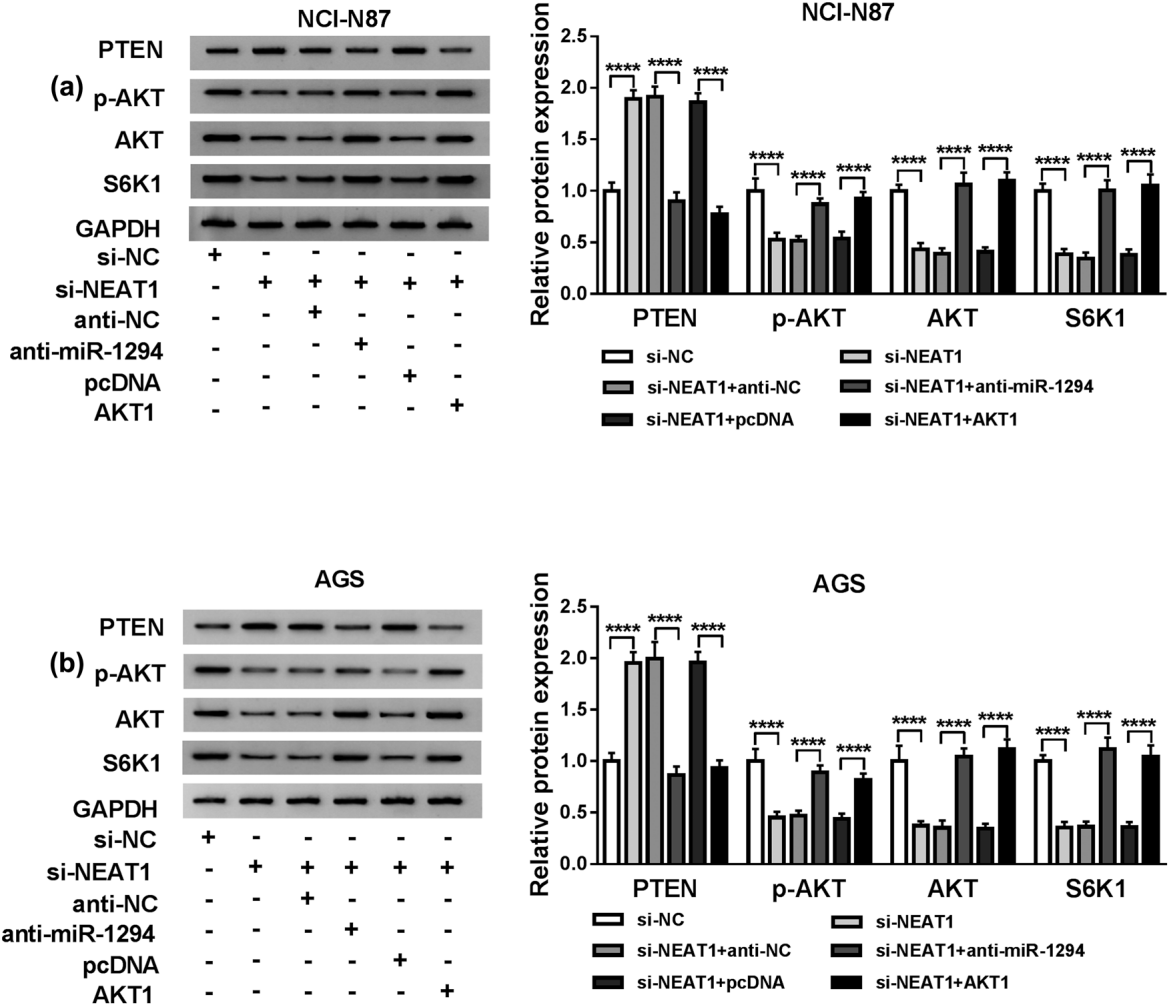
Down-regulation of NEAT1 repressed PI3K/AKT/mTOR signaling pathway via elevating miR-1294 and decreasing AKT1. (a and b) Western blot was utilized to detect the expression levels of PTEN, p-AKT, AKT, and S6K1 in NCI-N87 cells co-transfected with si-NEAT1 + anti-miR-1294 or si-NEAT1 + AKT1 (a), as well as in AGS cells (b). ****P < 0.0001.

In order to explore whether PI3K/AKT/mTOR signaling pathway was affected by NEAT1, NCI-N87 and AGS cells were, respectively, transfected with si-NEAT1, si-NEAT1 + anti-miR-1294, si-NEAT1 + AKT1, and relative controls. Western blot indicated that si-NEAT1 conspicuously enhanced the expression of PTEN and repressed the levels of p-PKT, AKT, and S6K1 in NCI-N87 cells, suggesting the PI3K/AKT/mTOR pathway was restrained. Interestingly, miR-1294 inhibition or AKT1 overexpression rescued the si-NEAT1-induced effects on PTEN, AKT, and S6K1 (Figure 6a). Also, the same phenomenon was viewed in AGS cells (Figure 6b). All these results demonstrated that the down-regulation of NEAT1 inhibited PI3K/AKT/mTOR signaling pathway via regulating miR-1294 and AKT1 in GC cells.

## 4 Discussion

As one of the most threatening malignancies for human health, the treatment of GC faces enormous challenges due to its difficult diagnosis and poor prognosis. It is imperative to search for novel biomarkers and explore the action mechanism underpinning GC. In our report, NEAT1 was found to act as an oncogene by activating the PI3K/AKT/mTOR pathway via modulating the miR-1294/AKT1 axis, providing novel evidence for NEAT1 as a diagnostic and therapeutic marker in the clinical treatment of GC.

LncRNA NEAT1 was manifested to participate in different biological processes of cancers. For example, Qian et al. found that NEAT1 inhibited cell growth of breast cancer by affecting miR-101-mediated EZH2 expression [17], and Zhen et al. clarified that knockdown of NEAT1 repressed glioma cell growth and invasion via targeting miR-107 to modulate CDK14 [36]. NEAT1 was also shown to promote colon cancer progression via up-regulating CDK6 by sponging miR-495-3p [10]. Recently, NEAT1 was reported to be up-regulated and knockdown of NEAT1 repressed migration and invasion of GC cells [3]. Zhang et al. also declared that silencing NEAT1 refrained GC cell proliferation, invasion, and induced apoptosis [34]. Consistent with these findings, NEAT1 expression was significantly elevated in our collected GC tissues and cultured cell lines. The functional exploration demonstrated that GC cell proliferation and metastasis were inhibited while apoptosis was expedited after knockdown of NEAT1, indicating the tumorigenic role of NEAT1 in GC progression.

LncRNAs were verified to exert function in cancer development by sponging miRNAs. For instance, TUBA4B was reported to inhibit GC progression by targeting miR-214 and miR-216a/b [8] and lncRNA H19 could regulate GC cell growth and metastasis via the sponge effect on miR-22-3p [4]. In our present report, we found miR-1294 had a low expression in GC tissues and cells, and there was an apparent negative correlation between NEAT1 and miR-1294. Their interaction was affirmed by dual-luciferase reporter assay, and NEAT1 generated the negative regulation of miR-1294 level. Further revert experiments manifested that knockdown of NEAT1 reduced proliferation, metastasis, and promoted apoptosis via up-regulating miR-1294 in GC cells.

Increasing studies have reported that miRNAs were involved in regulating the biological behaviors of GC by the regulation of downstream genes. MiR-671-5p and miR-1297 repressed proliferation and promoted apoptosis of GC cells, respectively, by targeting URGCP [18] and CDC6 [35]. Additionally, miR-664a-3p contributed to the evolution of GC via targeting the Hippo pathway [29]. To seek the target genes of miR-1294 in GC, Starbase v2.0 was used for target prediction and AKT1 was validated as a target gene of miR-1294. AKT1 was also up-regulated in GC, and NEAT1 was positively related to AKT1 in GC tissues. More interestingly, NEAT1 was shown to motivate AKT1 expression via sponging miR-1294. Zhou et al. found that NEAT1 promoted glioma progression by regulating the miR-132/SOX2 axis [37]. In this study, AKT down-regulation was accountable for the si-NEAT1-mediated inhibition of proliferation, migration, invasion, and motivation of apoptosis in GC cells, implicating that NEAT1 acted in GC biological regulation via the miR-1294/AKT1 axis. As previously reported in GC, NEAT1 promoted cellular proliferation, migration, and invasion by regulating the miR-506/STAT3 axis [24] and miR-335/ROCK1 axis [28]. In addition, Xia et al. have clarified that NEAT1 enhanced proliferation while blocked apoptosis of GC cells through elevating PIK3R1 as a sponge of miR-497-5p [32]. Specifically, we affirmed the existence of NEAT1/miR-1294/AKT1 in NEAT1-based GC pathomechanism different from the past.

Recently, dysregulated lncRNAs have been presented to be implicated in the malignant progression of GC via affecting the PI3K/AKT/mTOR signaling pathway [39]. For example, lncRNA LOC101928316 facilitated the development of GC by modulating the PI3K/AKT/mTOR signaling pathway [12]; MALAT1/miR-183/SIRT1 axis contributed to the regulation of GC via PI3K/AKT/mTOR pathway [13] and lncRNA XLOC_006753 could induce cell proliferation, cell cycle, and metastasis but inhibit apoptosis in multidrug-resistant GC cells through promoting the PI3K/AKT/mTOR pathway [33]. NEAT1 has reported to activate the PI3K/AKT pathway to facilitate GC cell viability and migration [27]. In this study, our western blot results revealed that knockdown of NEAT1 promoted the level of inhibitory protein PTEN while refrained the levels of p-AKT, AKT, and S6K1 in the PI3K/AKT/mTOR pathway. Given the function restoration of si-NEAT1 by miR-1294 inhibition or AKT1 elevation, it was considered that NEAT1 activated the PI3K/AKT/mTOR pathway by targeting the miR-1294/AKT1 axis. Thus, the NEAT1/miR-1294/AKT1/PI3K/AKT/mTOR signal network was disclosed first by our current study.

## 5 Conclusion

In conclusion, this report clarified that NEAT1 interacted with miR-1294 and indirectly promoted the expression of AKT1, thereby activating the PI3K/AKT/mTOR pathway to promote the progression of GC. Our outcomes laid a great foundation for understanding the functional mechanism of NEAT1 in GC and verifying NEAT1 as a useful biomarker in GC diagnosis and treatment.
